# Auxin treatment increases lifespan in *Caenorhabditis elegans*

**DOI:** 10.1242/bio.058703

**Published:** 2021-05-07

**Authors:** Julia A. Loose, Arjumand Ghazi

**Affiliations:** 1 Department of Pediatrics, University of Pittsburgh School of Medicine, Rangos Research Center, Children's Hospital of Pittsburgh, 15224 PA, USA; 2 Departments of Developmental Biology and Cell Biology and Physiology, University of Pittsburgh School of Medicine, Rangos Research Center, Children's Hospital of Pittsburgh, 15224 PA, USA

**Keywords:** *Caenorhabditis elegans*, Aging, Auxin, Lifespan, Protein degradation, Stress

## Abstract

The auxin-inducible degradation system (AID) has proven to be a highly versatile technology for rapid, robust and reversible depletion of proteins in multiple model systems. In recent years, AID has been adapted into the nematode *Caenorhabditis elegans* as a tool for conditional protein knockdown. Numerous transgenic strains have been created that, upon auxin exposure, undergo protein inactivation in the worm germline or somatic tissues, both during development and in young adults. Since longevity assays often involve long-term gene- and protein-manipulation, the facility for spatiotemporally precise and extended protein removal makes AID a potentially highly valuable tool for aging biology. However, whether auxins themselves impact worm longevity has not been directly addressed. Here, we show that prolonged exposure to indole 3-acetic acid (IAA), the auxin used in worm AID studies, extends lifespan. We also report that two transgenic strains expressing *Arabidopsis* proteins that are key components of the AID platform are longer lived than wild-type animals. Together, our results highlight the necessity for exercising caution while utilizing AID for longevity studies and in interpreting the resulting data.

This article has an associated First Person interview with the first author of the paper.

## Abstract (precis)

**Summary:** We report that auxin and auxin-inducible protein degradation tools alter lifespan in *C. elegans.*

## INTRODUCTION

The ability to bring about spatiotemporally controlled manipulation of genes and proteins is essential for elucidating biological mechanisms in any system. The nematode, *Caenorhabditis elegans*, is a leading genetic model organism, in part, due to its amenability to numerous gene knockdown and protein expression technologies. Gene inactivation by RNA interference (RNAi) was first discovered in worms and ‘RNAi-by-feeding’ remains a popular means for gene depletion (Fire, et al., 1998). A host of other techniques have also been described for site- and stage-specific control of worm gene expression, including the Q-system, the FLP-FRT platform and tissue-specific expression with TALEN nucleases (Nance and Frøkjær-Jensen, 2019). However, many of these approaches are irreversible and/or target mRNAs rather than proteins. In particular, concerns of indiscriminate gene inactivation and unintentional silencing of ‘off-target’ genes have revealed the limitations of feeding RNAi experiments (Rual et al., 2007). Several mutants and genetically engineered strains have been created to subvert these inadequacies and allow RNAi selectively within the germline or soma, or in specific cells types such as neurons (Calixto, et al., 2010; Qadota, et al., 2007). However, emerging evidences indicate that these strains are often ‘leaky’, limiting spatial precision (Kumsta and Hansen, 2012).

**Fig. 1.**
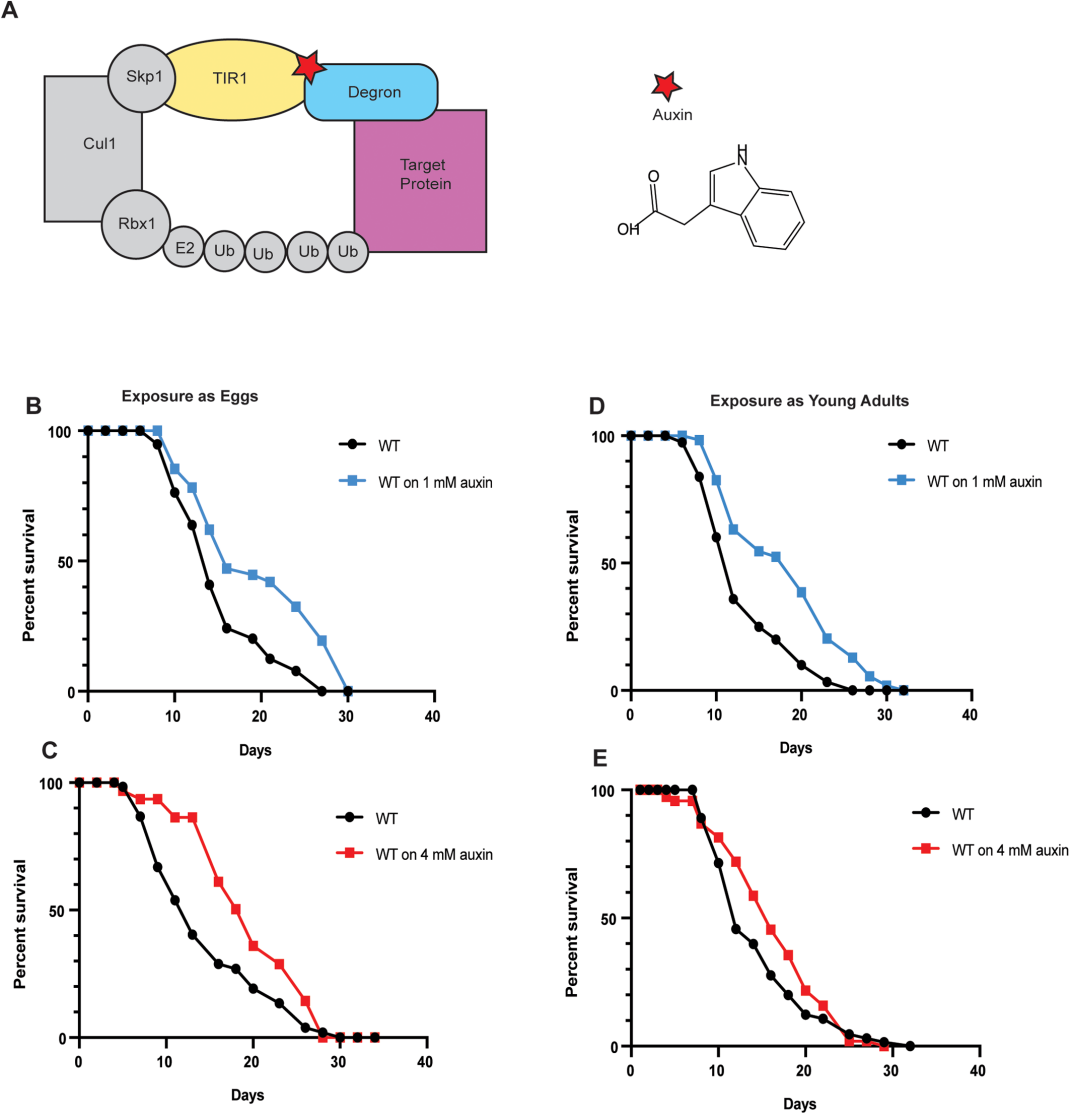
**Auxin exposure increases lifespan.** (A) Schematic representation of the auxin degradation system. (B–E) Lifespans of wild-type (WT) *C. elegans* exposed to 1 mM (blue, B, D) or 4 mM (red, C,E) IAA starting at embryonic stage (B, C) or as pre-adult L4 (D, E). (B) 1 mM auxin, Egg exposure: Control (m=15.25±0.58, *n*=75/123), 1 mM auxin (m=19.64±0.81, *n*=69/120) *P*>0.0001. (C) 4 mM auxin, Egg exposure: Control (m=14.54±0.89, *n*= 54/67), 4 mM auxin (m=19.46±1.15, *n*=28/45) *P*=0.0083. (D) 1 mM auxin, L4 exposure: control (m=13.4±0.49, *n*=104/118), 1 mM auxin (m=18.35±0.51, *n*=91/125) *P*>0.0001. (E) 4 mM auxin, L4 exposure: control (m=14.64±0.66, *n*=71/101), 4 mM auxin (m=16.43±0.78, *n*=53/90) *P*=0.1343. Survival and lifespan data shown as mean±standard error of the mean (s.e.m.). ‘n’ refers to number of worms analyzed over total number of worms tested in the experiment (see Materials and Methods for details). Statistical significance was calculated using the log rank (Mantel Cox) method. Data from additional trials in Table 1.

Recently, the auxin-inducible degradation (AID) system from plants has been introduced into *C. elegans* to enable conditional protein elimination (Zhang, et al., 2015). The plant AID relies on a protein, TIR1, the substrate recognition component of the Skp1-Cullin-FBox (SCF) E3 ligase complex. TIR1 is unique in binding substrates only in the presence of the phytohormone, auxin, before targeting them for proteasomal degradation (Gray, et al., 1999; Kepinski and Leyser, 2005; Ruegger, et al., 1998) (Fig. 1A). The auxin-dependent ability of TIR1 to degrade proteins even in heterologous systems such as yeast or mammalian cell lines has been harnessed to devise a highly robust, conditional protein knockdown strategy (Holland, et al., 2012; Nishimura, et al., 2009). The AID system was adapted into *C. elegans* by introducing a red fluorescent protein (mRuby)-linked *Arabidopsis* TIR1 protein under control of promoters and 3′ untranslated regions (UTRs) that direct expression selectively in the germline or in somatic tissues (Zhang, et al., 2015). To target specific worm proteins for TIR1 mediated degradation, Zhang et al., tagged them with a 44 amino acid degron sequence, derived from *Arabidopsis thaliana* IAA17 protein, as well as a synthetic GFP transgene for visualizing protein depletion (Morawska and Ulrich, 2013; Zhang, et al., 2018; Zhang, et al., 2015). In animals carrying both the transgenes, exposure to auxin causes TIR1 to bind the degron-tagged endogenous target protein and destine it for proteasomal degradation.

**Table 1.**
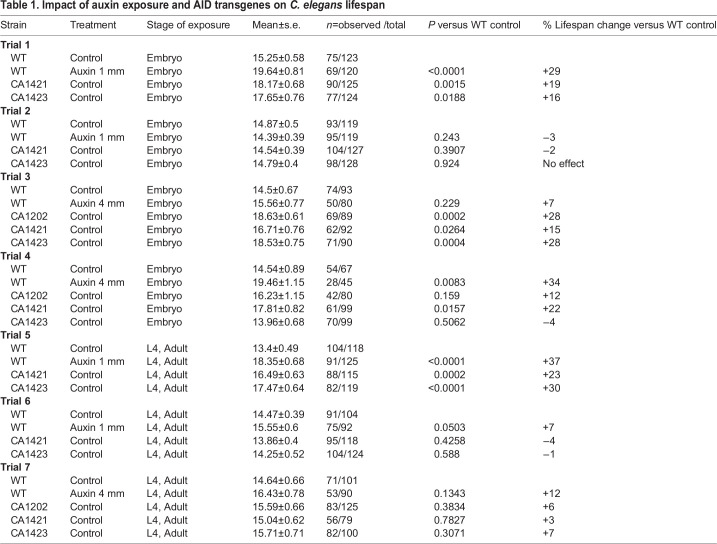
Impact of auxin exposure and AID transgenes on *C. elegans* lifespan

Auxins are critical phytohormones that regulate gene expression in a wide spectrum of biological processes in plants. The most common natural auxin, indole-3-acetic acid (IAA), is derived from the essential amino acid, tryptophan, in all organisms (Teale, et al., 2006). Indeed, intestinal commensal bacteria are known to metabolize tryptophan to produce indole and indole-related bioactive compounds (Roager and Licht, 2018). So far, exogenous IAA has been the most commonly used auxin in worm AID studies, although use of an indole-free, synthetic auxin, 1-napthaleneacetic (NAA), has been described recently (Martinez, et al., 2020). In their preliminary analyses, Zhang et al. showed that embryos grown on 1 mM or 4 mM IAA exhibited a normal developmental rate. Hermaphrodite mothers laid a normal brood size on IAA plates at 20°C, although, at 25°C, brood size was moderately diminished on 4 mM IAA. They also reported normal developmental rates and brood size in the TIR1 and degron expressing transgenic strains (Zhang, et al., 2015). However, whether IAA exposure itself impacts longevity, especially upon long-term treatment, has not been directly tested. This is an especially pertinent question for the aging field as longevity assays often require gene or protein knockdown to be sustained for at least a substantial fraction of the 2–3 week lifespan of worms. Indeed, in a recent report, a strain that expresses TIR1 in the worm germline to facilitate AID-mediated sterility was found to have longer median and maximum lifespans when exposed to IAA during the pre-adult (L4 larval) stage (Kasimatis, et al., 2018). Importantly, indoles from commensal bacteria have been shown to impact lifespan and health span in worms and other species (Sonowal, et al., 2017). A recent study also found that IAA increases resistance against endoplasmic reticulum (ER) stress-induced unfolded protein response (UPR^ER^) in worms (Bhoi, et al., 2021). In light of the strong correlation between longevity and stress resistance (Zhou, et al., 2011), this observation raises questions on possible effects of auxins themselves on worm lifespan.

While exploring the utility of the AID strains in studying how germline integrity impacts organismal longevity, we made the serendipitous discovery that long-term IAA treatment lengthened worm lifespan. Initiating IAA exposure from embryonic stages onwards, or commencing it on the cusp of adulthood at the L4 larval stage, produced persistent lifespan extensions in wild-type *C. elegans* across multiple trials. Additionally, we also found that at least two of three transgenic AID strains expressing *Arabidopsis* TIR1 and degron tags we tested exhibited longer lifespans than wild-type controls. Since longevity assays often necessitate extended gene and protein downregulation, our results underscore a need for exercising caution while utilizing AID technology for longevity studies, and in interpreting the data resulting from their usage.

## RESULTS

### Auxin exposure during development extends *C. elegans* lifespan

AID-dependent protein degradation in worms utilizes a transgenic strain that expresses the degron-tagged target protein as well as fluorescent-labeled TIR1 in the tissue of choice (Fig. 1A). Protein depletion is initiated by exposing the transgenic worms to auxin via transfer to IAA-supplemented worm growth (NGM-Agar) plates. Previously, Zhang et al. reported that exposure to 1 mM IAA is sufficient to bring about depletion of nuclear and cytoplasmic proteins in the worm germline within a 20 min to 2 h time frame without causing toxicity. Up to 4 mM IAA has been used as well without detrimental effects on developmental time or fecundity (Zhang, et al., 2015). In order to test if the AID system can be used for long-term depletion of proteins often necessitated for lifespan studies, we conducted control experiments in which we exposed wild-type animals from embryonic stage onwards to 1 mM or 4 mM IAA. Worms were transferred to fresh IAA plates every 48 h till at least Day 5 of adulthood (that signifies middle age and near-cessation of reproductive activity). We found that exposure to either 1 mM or 4 mM IAA significantly increased lifespan in three of four trials, two of which were highly statistically significant (29% and 34%, respectively) (Fig. 1B,C). Transferring worms to fresh IAA plates every day, or extending exposure to freshly made IAA plates till Day 12 of adulthood did not further enhance longevity (Table 1). Thus, sustained auxin exposure during development increased worm lifespan.

### Auxin exposure during adulthood increases *C. elegans* lifespan

To test if the impact of auxin treatment was due to developmental intervention (and since aging studies often require gene inactivation in adult animals), we restricted IAA exposure solely to post-developmental stages and assessed the lifespan of worms. L4-staged larvae were introduced to 1 mM or 4 mM IAA plates on the cusp of adulthood, and transferred to fresh IAA-laced plates every other day. Lifespan was extended significantly upon 1 mM IAA treatment (by 37% and 7%, respectively) (Fig. 1D, Table 1). 4 mM treatment caused a 12% lifespan extension but did not achieve statistical significance (Fig. 1E, Table 1). As with developmental exposure, increasing the frequency of transfers to fresh IAA plates (every 24 h) or extending exposure on fresh IAA plates beyond Day 5 of adulthood did not further augment lifespan (Table 1). Thus, the longevity-extending impact of IAA is pervasive; it is not restricted to developmental or adult stage of exposure, and is observed at the lowest auxin concentration commonly used for AID studies.

### Strains facilitating AID-mediated germline protein depletion are long lived

**Fig. 2.**
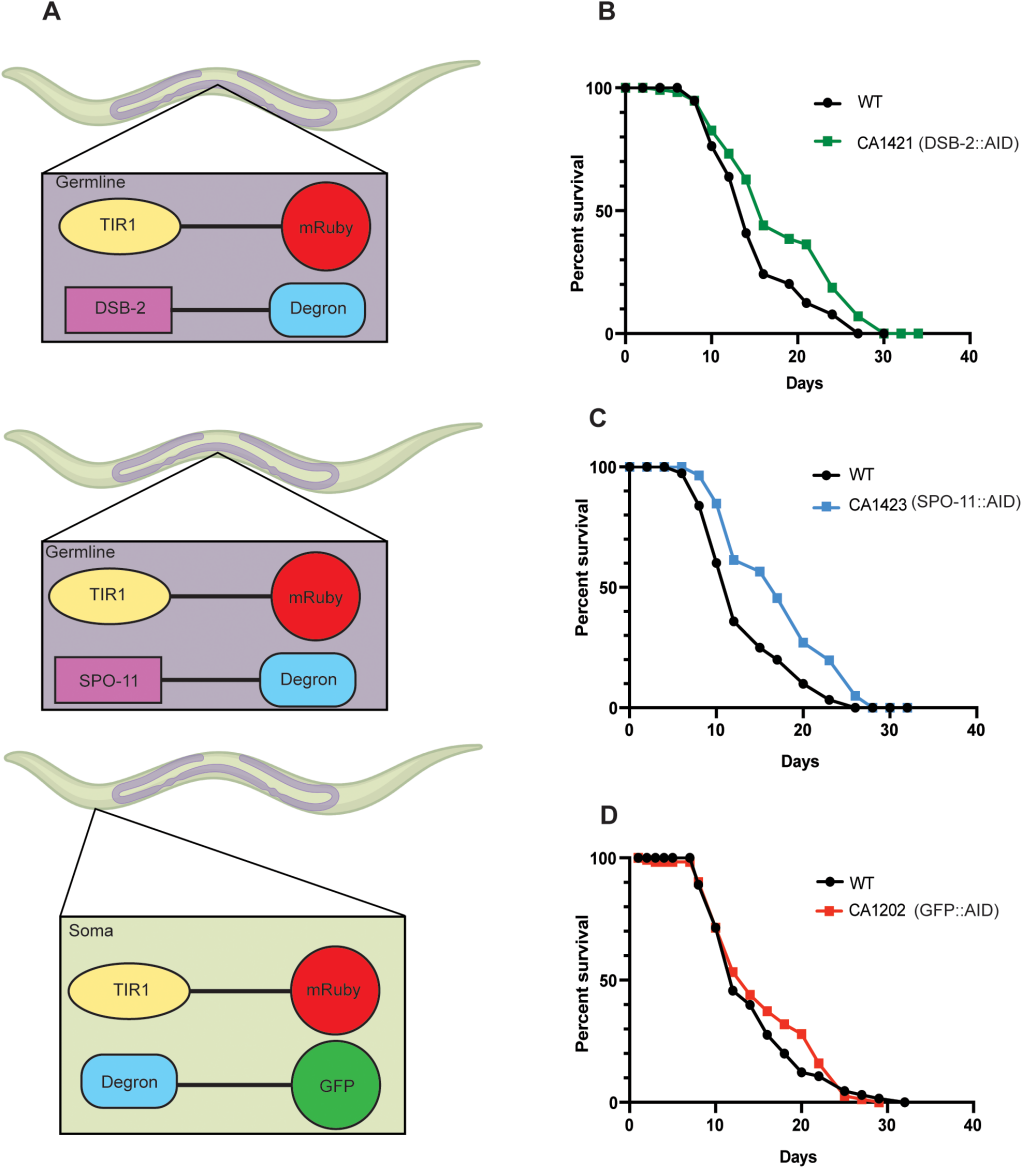
**Strains expressing TIR1 and degron tagged proteins extend lifespan.** (A) Schematic representation of AID strains tested in the study. Transgenic animals express TIR1 in either the germline (CA1421 and CA1423, top and middle panel) or soma (CA1202, bottom panel) along with indicated degron tagged proteins in each. Corresponding lifespan curves shown in B. (B) Wild type (WT): m=15.25±0.58, *n*=75/123, CA1421: m=18.17±0.68, *n*=90/125, *P*=0.0015. (C) WT: m=13.4±0.9, *n*=104/118, CA1423: m=17.47±0.64, *n*=88/115, *P*>0.0001. (D) WT: m=14.64±0.66, *n*=71/101, CA1202: m=15.59±0.66, *n*=83/125, *P*=0.3834). All lifespans conducted on NGM plates containing 2.5% ethanol +/− IAA. Survival and lifespan data shown as mean±standard error of the mean (s.e.m.). ‘n’ refers to number of worms analyzed over total number of worms tested in the experiment (see Materials and Methods for details). Statistical significance was calculated using the log rank (Mantel Cox) method. Data from additional trials in Table 1. Fig. 2A created using BioRender.com.

The widespread use of AID technology in worms has been facilitated by the generation of engineered strains that express TIR1 under control of promoters and 3′ UTRs driving expression in specific tissues. Zhang et al., who first developed the system in worms, have created a repertoire of such strains and used them to investigate the roles of germline proteins in crossover control during meiosis (Zhang, et al., 2018). Due to our interest in the reproductive control of aging, we tested the longevity of two of these strains. Both express mRuby-tagged TIR1 under control of the promoter and 3′UTR of *sun-1 (Psun-1::TIR1::mRuby)* (Fig. 2A). *sun-1* is expressed in all mitotic and meiotic germline cells as well as proliferating embryonic cells so the transgene is an effective means of eliminating proteins in the germline and early embryos (Minn, et al., 2009). In addition, these strains carried the *spo-11* locus (CA1423) or *dsb-2* locus (CA1421) tagged with the degron sequence and a 3xFLAG epitope (Fig. 2A). *spo-11*, encoding a conserved topoisomerase, and *dsb-2*, encoding a worm-specific double-stranded break protein, are key mediators of double strand DNA break formation during meiosis, with predicted germline restricted expression. The third strain, CA1202, expresses mRuby-tagged TIR1 as well as the GFP-linked degron under control of a promoter of *eft-3* that shows universal somatic expression. This strain has been used as a control to monitor AID-dependent somatic protein degradation (Zhang, et al., 2015). We found two of the three strains to be significantly longer lived than wild-type animals under basal control conditions even without IAA exposure (Fig. 2, Table 1). In five of seven trials, CA1421 exhibited a significant lifespan extension, ranging from 3% to 23%, compared to the wild-type control grown on solvent alone (four of five achieved statistical significance) (Fig. 2B, Table 1). CA1423 was long lived in five of seven trials as well (7% to 30% increase) of which three were statistically significant (Fig. 2C, Table 1). The third strain, CA1202, showed a 6% to 28% lifespan extension in all three trials conducted of which one was statistically highly significant (Fig. 2D, Table 1). These results indicate that, in at least two instances, transgenic expression of *Arabidopsis* TIR1 protein and degron in *C. elegans*, especially within the germline, is sufficient to enhance lifespan irrespective of auxin supplementation.

## DISCUSSION

In this study, we show that two key components of the AID conditional protein deletion technology impact *C. elegans* lifespan. Chronic exposure to IAA, currently the most widely used auxin in AID studies, begun either during development or young adulthood, increased the lifespan of worms. Further, at least two transgenic AID strains expressing heterologous TIR1 and AID-degron proteins exhibited longer lifespans per se even in the absence of IAA. Though these effects are modest and vary between trials, they suggest a strong need for caution while utilizing existing AID reagents for longevity analyses and interpreting the resulting data.

The IAA-mediated lifespan extension we observed here is partly in accordance with studies on the impact of indoles, the precursors of auxins, on *C. elegans* health and longevity. Recent studies have reported the effects of altering indole levels, either through bacterial mutants or media supplementation, on lifespan and health span in flies and worms but with varying results (Lee, et al., 2017; Sonowal, et al., 2017). Lee et al. found indole supplementation at high concentration to shorten worm lifespan in a dose dependent manner, 1 mM Indole in fact reduced lifespan by 12 days (Lee, et al., 2017). However, Sonowal et al. reported lower indole concentrations (100 µM) to improve health span measures (Sonowal, et al., 2017). Lifespan differences have also been observed between worms grown on indole-producing versus indole-deficient bacterial strains (Lee, et al., 2017; Sonowal, et al., 2017). Interestingly, Lee et al. also observed specific effects of supplementation with some indole derivatives: 2-Oxindole shortened worm lifespan, whereas, N-β-D-glucopyranosyl-indole, produced no change (Lee, et al., 2017). Thus, the effects of altering indoles are nuanced and dependent upon aspects of host and bacterial physiology. It is likely that IAA supplementation impinges on similar pathways. Since our studies were conducted using live *Escherichia coli*, it is not clear if the effects we saw are attributable to bacterial or host metabolism. It is also noteworthy that auxins have been found to play roles in plant responses to biotic and abiotic stressors, in part through modulation of reactive oxygen species (ROS) (Krishnamurthy and Rathinasabapathi, 2013). ROS are integral to aging mechanisms and the close relationship between stress resistance and longevity has been well established (Ristow and Schmeisser, 2011). Pertinently, a recent report demonstrated that IAA supplementation enhanced UPR^ER^ stress resistance in worms (Bhoi, et al., 2021). So, it is possible that IAA-mediated lifespan extension acts through ROS and stress-response pathways.

The AID system has also been utilized to create a strain, PX627, that undergoes inducible sterility by auxin-mediated degradation of a spermatogenesis protein, SPE-44 (Kasimatis, et al., 2018). In PX627, TIR1 expression is driven in the germline using the germ-cell specific *pie-1* promoter*.* PX627 has been characterized for mitochondrial and health span parameters but mainly in comparison to the effects produced by fluoro-deoxy uridine (fUDR), an alternative sterility-induction treatment (Dilberger, et al., 2020). Dilberger et al. found PX627 to show similar lifespan upon supplementation with 1 mM auxin versus FUdR (Dilberger, et al., 2020). However, Kasimatis et al. found that the strain exhibited lower late life mortality on auxin as compared to unsupplemented animals (Kasimatis, et al., 2018). PX627 adults were also reported to exhibit remarkably greater mobility during old age (Dilberger, et al., 2020). These observations merit attention because two of the AID transgenic strains we examined also express TIR1 within the germline albeit using a different promoter, and degron tags associated with germline proteins (Zhang, et al., 2018). This raises the possibility that expression of heterologous *Arabidopsis* proteins, especially in the worm germline, may have unintended lifespan consequences. We also noted that CA1432 animals, wherein *spo-11* inactivation is expected to produce embryonic lethality (Dernburg, et al., 1998; Rosu, et al., 2013), produced dead embryos in early adulthood, but by Day 4, embryonic lethality was considerably diminished and larvae could be observed crawling on the plates. Similarly, the GFP signal in the CA1202 strain was initially eliminated upon commencing IAA exposure as expected, but recovered after a few days (∼Day 8) (data not shown) suggesting that target protein knockdown may not be sustained over time despite continued IAA supplementation, and the organism may deploy countermeasures to regain protein levels. This feature, along with reports of ‘leaky’ protein depletion observed in other systems, also needs to be considered in AID studies (Yesbolatova, et al., 2020) as new strains and reagents for worm AID studies are being generated rapidly (Ashley, et al., 2021). Fortuitously, recent versions of AID have been described that utilize a mutant TIR1 that prevents leaky degradation and a synthetic ligand 5-phenyl-indole-3-acetic acid (5-Ph-IAA), that activates TIR1 at a concentration 670 times lower than those currently used for IAA (Yesbolatova, et al., 2020). Introducing these features into worm AID platforms are likely to enhance the utility of the technology greatly. Meanwhile, our observations suggest that significant caution is required in employing existing AID reagents for longevity studies.

## MATERIALS AND METHODS

### *Caenorhabditis elegans* maintenance

Strains were maintained at 20°C on nematode growth media (NGM) plates seeded with *E. coli* strain OP50. Strains used include N2 (wild type), and the following strains obtained from the CGC, CA1421 (*meIs8 [pie-1p::GFP::cosa-1+unc-119(+)]II;dsb-2(ie58[dsb-2::AID::3xFLAG] ieSi38 [sun-1p::TIR1::mRuby::sun-1 3′UTR +Cbr-unc-119(+)] IV*, CA1423 *(meIs8 [pie-1p::GFP::cosa-1+unc-110(+)]II; spo-11(ie59[spo-11::AID::3xFLAG]) ie Si38, [sun-1p::TIR1::mRuby::sun-1 3′UTR +Cbr-unc-119(+)]* IV and CA1202 (*ieSi57 [eft3p::TIR1::mRuby::unc-54 3′UTR+Cbr-unc119(+)] II; ieSi58 [eft-3p::degron::GFP::unc-54 3′UTR+Cbr-unc-119(+)] IV.*

### Lifespans assays

For the lifespan assays, either eggs or L4 animals were placed on auxin (1 mM or 4 mM) or the ethanol (2.5%) solvent alone. Indole-3-acetic acid from Alfa Aesar (A#10556) was dissolved in ethanol and filter sterilized before adding to cooled NGM media before plates were poured. The lifespan assays were conducted at 20°C, with L4 stage marked as Day 0. Animals were observed every other day unless otherwise indicated and transferred to new plates every other day during the reproductive period unless otherwise noted. Animals were categorized as dead, alive or censored (bagged or missing). The lifespan assays were analyzed using the program Online Application of Survival Analysis 2 (OASIS 2) to calculate *P*-values (Han, et al., 2016). GraphPad Prism (version 9) was used to graph the results.
